# Books and Pamphlets Received

**Published:** 1888-09

**Authors:** 


### BOOKS AND PAMPHLETS RECEIVED.

*A System of Obstetrics by American Authors.* Edited by Barton Cook Hurst, M. D., Associate Professor of Obstetrics in the University of

Pennsylvania; Obstretician to the Philadelphia and Maternity Hospital; Gynecologist to the Orthopædic Hospital; Fellow of the College of Physicians of Philadelphia, etc. Vol. I. Illustrated with a colored plate and 309 wood engravings. Philadelphia: Lea Brothers & Co. 1888.

This is the first volume of a large and comprehensive treatise on obstetrics, designed to embody the knowledge of the best writers and specialists on sub jects embraced in this important department. The book will be large, the present volume numbering over 800 large octavo pages. The obstetrician, it is expected, will find in the work, when complete, a guide for all conditions and emergencies that can arise. The following are the names of the contributors to the present volume, to wit: Samuel C. Busey, M. D., Washington, D. C.; Geo. D. Englemen, M.D , St. Louis; Barton Cook Hurst, M.D., Philadelphia; William Wright Jaggard, A.M., M.D., Chicago; H. Newell Martin, M. D., D. Sc. M.A., Baltimore; Theophilus Parvin, M.D., LL. D., Philadelphia; R. A. F. Penrose, M. D., LL. D., Philadelphia; J. C. Reeve, M. D., Dayton.

The subjects treated are as follows: "The History of Obstetrics;" the "Physiology and Histology of Ovulation, Menstruation and Fertilization, the Development of the Embryo;" "Pregnancy, its Physiology, Pathology, Signs and Differential Diagnosis;" the "Conduct of Labor and the Management of the Puerperal State;" on the "Mechanism of Labor, and the Treatment of Labor based on the Mechanism;" the "Use of Anesthetics in Labor;" "Anomalies of the Forces in Labor."

We regard this work as the most able and elaborate we have seen in the department of obstetrics, and which should be in the library of every progressive man in the profession.

*The Practice of Medicine and Surgery Applied to the Diseases and Accidents Incident to Women.* By W. H. Byford, A. M., M. D., Professor of Gynecology in Rush Medical College, and of Obstetrics in the Women's Medical College; Surgeon to the Women's Hospital of Chicago; ex-President of the American Gynecological Society; ex-Vice-President of the American Medical Association, etc., and Henry T. Byford, M.D., Surgeon to the Woman's Hospital; President of the Gynecological Society; Member of American Medical Association, Illinois State Medical Society, Chicago Medical Society, etc. Fourth edition revised, rewritten and very much enlarged with 306 illustrations. Philadelphia: P. Blakiston, Son & Co. 1888. 832 pp. Cloth, $5.00; leather, $6.00.

We have here a very large and ably gotten up book on "Medicine and Surgery Incident to Women." The style and matter of the work is excellent. The illustrations are mostly new and admirably drawn. The Anatomy, Physiology and topography of the pelvic organs are ably taught and discussed, and advanced thoughts are presented upon all important subjects connected with gynecology.

The chapters on "Lacerations of the Uterus, and Affections of the Perineum, Displacements of the Uterus, and Affections of the Uterus" are especially full and instructive. So, also, remarks on "Fallopian Tubes," "Oophorectomy," "Ovarian Tumors," "Abdominal Ovariotomy,', etc.

The subjects are ably and scientifically treated and brought down to the latest advances.

*A Manual of the Minor Gynecological Operations.* By J. Halliday Croom, M. D., F. R. C. P. E., Lecturer on Midwifery and the Diseases of Women at the School of Medicine; Physician to the Royal Maternity Hospital; Physican for Diseases of Women Western Dispensary; Vice President of the Obstetrical Society. Edinburg. First American from the second Edinburg edition revised and enlarged. By Lewis McMurty, A.M., M.D., formerly Professor of Anatomy in the Kentucky School of Medicine; Member of the Medical Society of Louisville; First Vice President of the Kentucky State Medical Society; Member of the Boyle County Medical Society; Corresponding Member of the Obstetrical Society of Philadelphia. Records, McMullin & Co. 1888.

A work of 228 pp., neatly bound in cloth. The book "is intended to furnish the student and practitioner a brief, simple, and practical account of the more common gynecological operations." The operations and manipulations are explained in a brief and simple manner so as to be readily understood, and the improved apparatus and proceedure of late years are brought under review. The illustrations are good, and we regard the work as an eminently practical and useful one.

*Practical Electro Therapeutics.* By William F. Hutchinson, M.D., Philadelphia. Records, McMullin & Co. 1888.

We have here a compact work of 248 pages, the result as the author tells us of fifteen years of labor in the special domain of electricity as employed in medicine, and especially designed to meet the inquiries which constantly come up from the profession to how to use electricity in different diseases. The work is strictly practical and contains only such illustrations as presents a few instruments of his own invention. Static electricity is but lightly referred to for the reason that the author has found in it no better results than from faradism, which is more simple and better adapted to the general practitioner. The chapter upon electro surgery, we think, will prove very useful to practitioners who wish to test for themselves electricity as applied to galvano-cautery and electrolysis.

